# Enzyme Storage and Recycling: Nanoassemblies of α-Amylase and Xylanase Immobilized on Biomimetic Magnetic Nanoparticles

**DOI:** 10.1021/acssuschemeng.0c08300

**Published:** 2021-03-09

**Authors:** Karima Salem, Ylenia Jabalera, Jose David Puentes-Pardo, Jesus Vilchez-Garcia, Adel Sayari, Aïda Hmida-Sayari, Concepcion Jimenez-Lopez, Massimiliano Perduca

**Affiliations:** † Centre de Biotechnologie de Sfax (CBS), Université de Sfax, Route de Sidi Mansour Km 6, BP “1177”, 3018 Sfax, Tunisie; ‡ Departamento de Microbiologia, Universidad de Granada, Campus de Fuentenueva s/n, 18071 Granada, Spain; § ENIS, Université de Sfax, BP “1173”, 3038 Sfax, Tunisie; ∥ Department of Biotechnology, University of Verona, Strada Le Grazie 15, 37134 Verona, Italy

**Keywords:** immobilization, biomimetic magnetic nanoparticles, α-amylase, xylanase, storage stability, reusability

## Abstract

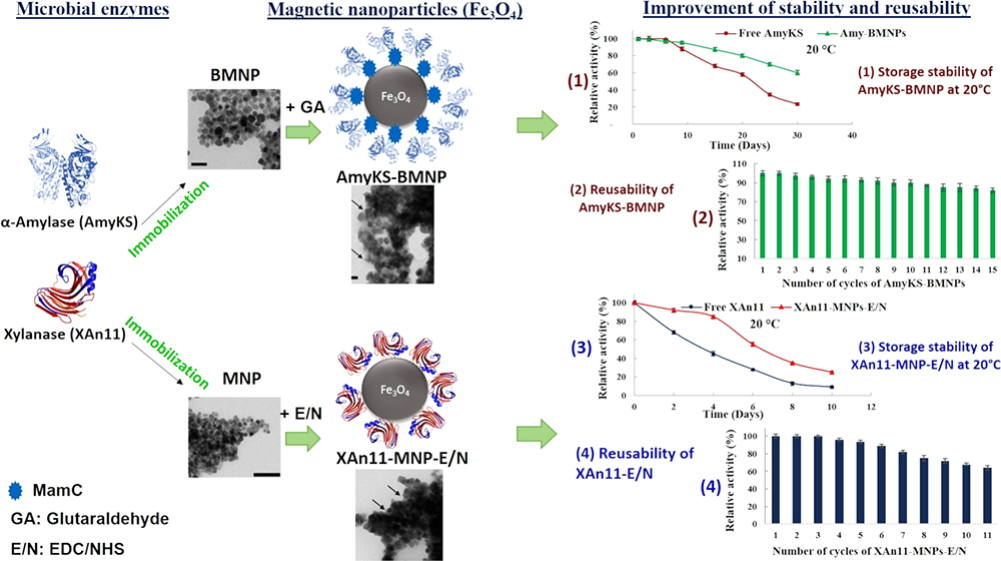

Immobilization of enzymes has been extensively required in a wide variety of industrial applications as a way to ensure functionality and the potential of enzyme recycling after use. In particular, enzyme immobilization on magnetic nanoparticles (MNPs) could offer reusability by means of magnetic recovery and concentration, along with increased stability and robust activity of the enzyme under different physicochemical conditions. In the present work, microbial α-amylase (AmyKS) and xylanase (XAn11) were both immobilized on different types of MNPs [MamC-mediated biomimetic MNPs (BMNPs) and inorganic MNPs] by using two different strategies (electrostatic interaction and covalent bond). AmyKS immobilization was successful using electrostatic interaction with BMNPs. Instead, the best strategy to immobilize XAn11 was using MNPs through the hetero-crosslinker 1-ethyl-3-(3-dimethylaminopropyl) carbodiimide (EDC) and *N*-hydroxysuccinimide (NHS). The immobilization protocols were optimized by varying glutaraldehyde (GA) concentration, enzyme quantity, and reaction time. Under optimal conditions, 92% of AmyKS and 87% of XAn11 were immobilized on BMNPs and MNPs–E/N, respectively (here referred as AmyKS–BMNPs and XAn11–MNPs nanoassemblies). The results show that the immobilization of the enzymes did not extensively alter their functionality and increased enzyme stability compared to that of the free enzyme upon storage at 4 and 20 °C. Interestingly, the immobilized amylase and xylanase were reused for 15 and 8 cycles, respectively, without significant loss of activity upon magnetic recovery of the nanoassemblies. The results suggest the great potential of these nanoassemblies in bioindustry applications.

## Abstract 2

In the present work, microbial α-amylase and xylanase were both immobilized on different types of MNPs by using two different strategies.

## Introduction

Enzymes are of great importance in industries because they are considered a good alternative to replace harmful chemical products.^1,2^ Indeed, these biocatalysts have remarkable properties, such as their excellent selectivity and high activity toward substrates that facilitate the most complex chemical processes by eliminating “bottlenecks” in chemical reactions.^3^

α-Amylases are one of the key enzymes in many industrial applications, able to catalyze the cleavage of α-d-1,4 glucosidic bonds of starch molecules to yield dextrin and other smaller glucose polymers. Applications range from the food industry and brewing, detergents, textile, biomass production to pharmaceutical and clinical chemistry.^4,5^ Xylanases (EC 3.2.1.x) are glycosidases which catalyze the endohydrolysis of β-1,4-xylosidic linkages in xylan. They belong to the glycoside hydrolase family, one of the largest groups of commercial enzymes^6^ for application in the food industry, pulp and paper, and biofuel, among others.^5^

Amylases and xylanases have some drawbacks that limit their use at the industrial scale, that is, their high sensitivity to extreme conditions, their low stability and their difficulty for recovery and recycling, influencing the production costs. Different strategies such as genetic and protein engineering and immobilization have been proposed to improve the activity and stability of these enzymes to increase their survival through technological processes.^7,8^ In particular, enzyme immobilization is one of the most efficient techniques exploited over the last decades for its efficient application at the industrial scale.^9,10^ In most immobilization procedures, the enzyme is fixed to the substrate via cross-linking.^11^

Both the type of binding and the substrate need to be accounted to improve the performance of the enzyme. Covalent bonds usually ensure longer-lasting (and stronger) nanoassemblies. However, the structure of the enzyme (and its stability) is often compromised, and the activity of the nanoassembly is fairly lower than that of the free enzyme.^12,13^ On the other hand, the electrostatic bond generally better maintains the structure of enzyme, but the enzyme is prone to detach early from the substrate, thus preventing an efficient enzyme recovery.^14^

In the context of the substrate, the immobilization of the enzyme on magnetic nanoparticles (MNPs) is raising interest due to their high specific surface area and easier separation from the reaction mixture using an external magnetic field.^15−17^ The most commonly used magnetic particles are magnetite (Fe_3_O_4_) and maghemite (c-Fe_2_O_3_) due to their low toxicity and biocompatibility.^18^ Most of commercial MNPs are superparamagnetic and display sizes below 20 nm and isoelectric point (pI) of ∼7.^19,20^ While being superparamagnetic is an advantage, since it prevents agglomeration of the MNPs due to dipole–dipole interaction in the absence of a magnetic field, the small size of these commercial MNPs makes them have, in many cases, low magnetic moment per particle that may result in failures responding to an external magnetic field. Moreover, most of them need to be coated with different molecules such as poly(ethylene glycol) or poly(vinyl alcohol) to provide them with functional groups needed for functionalization.^20,21^ This coating of the MNPs, on top of making the production process more expensive, shields the already nonoptimal magnetic moment per particle.^22^ Therefore, one way to improve enzyme stabilization, efficient recovery, and recycling is to improve the magnetic nanoparticle used as the substrate for immobilization.

Some of the drawbacks mentioned above may be corrected by taking an example from nature, that is, from the magnetosome formation in magnetotactic bacteria. In fact, magnetite nanoparticles produced by a magnetosome-associated protein (MamC from *Magnetococcus marinus* MC-1) are able to template the growth of MNPs *in vitro*, producing the so-called biomimetic MNPs (BMNPs) that are larger (∼40 nm) than most commercial MNPs, thus having a larger magnetic moment per particle.^23^ Also, MamC remains entrapped (or at least strongly attached) to the outer layers of the BMNPs, offering new surface properties (pI ≈ 4.4) and functional groups that make any further coating of the BMNPs unnecessary.

In the present work, the immobilization of a *Bacillus subtilis* α-amylase and an *Aspergillus niger* xylanase was attempted and optimized by using both electrostatic and covalent bond and two types of MNPs as substrates, both MNPs and MamC-mediated BMNPs. It is worth noting that this study is the first approach exploring the use of BMNPs to form enzymatic nanoassemblies for biotechnology purposes. While both types of nanoassemblies could be efficiently separated from the reaction mixture, the optimum results in terms of stability and activity of the nanoassembly were obtained when the recombinant α-amylase was attached to BMNPs using glutaraldehyde (GA) as the cross-linker and the recombinant xylanase was attached to MNPs mediated by 1-ethyl-3-(3-dimethylaminopropyl) carbodiimide (EDC)/*N*-hydroxysuccinimide (NHS) as the coupling agent. The influence of enzyme quantity, type of bond, and incubation time on the immobilization efficiency was determined. The successful immobilization led to an enhancement of enzyme stability following upon storage and to an easy recovery and reusability of the enzyme.

## Materials and Methods

### MNPs and MamC-Mediated BMNPs

The MNPs and BMNPs used in this study were synthesized and described by Jabalera et al. (2019).^24^ In brief, MamC cloning, expression, and purification were carried out as described by Valverde-Tercedor et al. (2015).^23^ MamC was purified under denaturing conditions by using a HiTrap chelating HP column (GE Healthcare) in an ÄKTA Prime Plus FPLC system (GE Healthcare) and then dialyzed to completely remove urea, allowing gradual MamC folding.

MNPs were obtained in closed systems containing the following master solution of Fe(ClO_4_)_2_ (2.78 mM), NaHCO_3_/Na_2_CO_3_ (3.5 mM/3.5 mM), and FeCl_3_ (5.56 mM), at pH 9, for 30 days, at 25 °C and 1 atm total pressure inside an anaerobic COY chamber to avoid potential oxidation. BMNPs were obtained under identical conditions by adding 10 μg/mL MamC. At the end of the reaction time, all precipitates were magnetically concentrated and the supernatant was removed. The pellet was suspended again in deoxygenated water and magnetically concentrated again. This rinsing procedure was repeated three times.

Powder X-ray diffraction analysis was carried out with an X’pert Pro X-ray diffractometer [PANalytical, Cu Kα-radiation, 20–60° in 2θ (0.01°/step; 3 s per step)]. Transmission electron microscopy (TEM) analyses were performed with a STEM Philips model CM20 microscope on ultrathin sections. The crystal size was measured on ∼1000 nanoparticles per experiment using ImageJ 1.47. As the BMNPs used in the present study belonged to the same batch as those characterized in Jabalera et al. (2019),^24^ basic mineral characterization is included in the present article, while further characterization (ζ-potential, thermogravimetric analyses and hysteresis cycle at 5 and 300 K) can be found in García Rubia et al. (2018)^20^ and Jabalera et al. (2019).^24^ According to these results, the MNPs and BMNPs used in the present study are superparamagnetic MNPs at 300 K, BMNPs composed of ∼95 wt % of magnetite and ∼5 wt % of MamC, being the isoelectric point (pI) for BMNPs of 4.4 and for MNPs of 7.2.

### Enzyme Preparation

An α-amylase from *B. subtilis* called AmyKS^25^ was expressed in *E. coli* and purified using Ni-NTA affinity chromatography.^26^ The purified fraction was concentrated by centrifugal filtration in a 50 kDa MW centrifugal filter unit (5000×*g*; 4 °C). The α-amylase solution was recovered in phosphate buffer (50 mM; pH 7) and stored at 4 °C until use. AmyKS is a dimeric protein with a molecular mass of around 140 kDa and pI of 5.61.

XAn11 is an *A. niger* xylanase expressed in *Pichia pastoris* and purified using Ni-NTA affinity chromatography.^26^ The purified fraction was concentrated by centrifugal filtration in a 10 kDa MW centrifugal filter unit (5000×*g*; 22 °C). The xylanase solution was recovered in citrate buffer (50 mM; pH 5) and stored at 4 °C until use. XAn11 is a monomeric protein with a molecular mass of around 24 kDa and pI of 4.31.

### Immobilization of AmyKS on BMNPs and MNPs

In order to immobilize AmyKS by means of electrostatic bond, 5 mg of MNPs in citrate buffer (pH 5, so the MNPs displayed a positive surface charge according to their pI) or 5 mg of BMNPs in *N*-(2-hydroxyethyl)piperazine-*N*′-ethanesulfonic acid (HEPES) buffer (pH of 7.4, so the BMNPs displayed a negative surface charge according to their pI) added with 1 M CaCl_2_ were mixed with the enzyme at a final concentration of 50 μM. The reaction mixture was incubated for 24 h at room temperature. Then, 5 μL of GA (25%) was added to the reaction and incubated one more hour at room temperature (Figure S1). The immobilized amylase (AmyKS–BMNPs) was recovered from the reaction mixture by a permanent magnet. After three wash steps with HEPES buffer + 150 mM CaCl_2_, the amount of immobilized AmyKS was indirectly determined by measuring the unimmobilized protein content in the supernatant using UV absorption spectroscopy at 280 nm.

Concerning the immobilization of AmyKS via EDC + NHS reaction, a mass of 5 mg of synthetized MNPs (MNPs or BMNPs) was suspended in 1 mL of 50 mM MES free oxygen buffer (pH 5.5) and activated with EDC (0.1 M) and NHS (0.7 M); the reaction solution was stirred for 1 h at 20 °C. Then, AmyKS, previously concentrated and washed with NaClO_4_ (pH 4.5), was added to the suspension at a final concentration of 50 μM and incubated for 24 h at room temperature. The coupling reaction was stopped by adding Tris 0.1 M, and then, the immobilized amylase (AmyKS–E/N) was magnetically recovered from the reaction mixture using a permanent magnet. After three wash steps with buffer NaClO_4_ (pH 4.5), the amount of AmyKS immobilized was determined as previously described. All experiments were run in triplicate.

### Immobilization of XAn11 on BMNPs and MNPs

The immobilization of XAn11 via electrostatic bonding was performed by mixing 25 μM of the enzyme washed previously with Tris buffer (pH 7) and mixed with either 5 mg of BMNPs in HEPES buffer at pH 7 or MNPs in citrate buffer at pH 5. The reaction mixture was incubated for 24 h at 4 °C. Then, 5 μL of GA (25%) was added to the reaction and incubated one more hour at room temperature. Also, the immobilized xylanase (XAn11–BMNPs) was magnetically recovered from the reaction mixture. After three wash steps with citrate buffer, the amount of xylanase immobilized was determined by measuring the free protein content in the supernatant using UV absorption spectroscopy at 280 nm.

For the immobilization of xylanase via EDC and NHS, 5 mg of synthetized MNPs (MNPs or BMNPs) was suspended in 1 mL of 50 mM MES free oxygen buffer (pH 5.5) and activated with EDC (0.1 M) and NHS (0.7 M) (Figure S2); the reaction solution was stirred 1 h at 20 °C. Then, 25 μM XAn11, previously concentrated and washed by NaClO_4_ (pH 3.5), was added to the suspension and incubated 24 h at 4 °C. The coupling reaction was stopped by adding Tris 0.1 M, and then, the immobilized xylanase (XAn11–MNPs–E/N) was magnetically recovered. After three wash steps with NaClO_4_, the amount of XAn11 immobilized was determined as previously described. All experiments were run in triplicate.

### Characterization of Immobilized Enzymes

#### ζ-Potential

These nanoassemblies that showed more efficient and stable binding (AmyKS–BMNPs and XAn11–MNPs–E/N) were selected for further characterization, as well as BMNPs and MNPs (as control samples). Stock suspensions of all samples were prepared in NaClO_4_ (10 mM). For each stock solution, seven vials were identically prepared by diluting the stock in NaClO_4_ (10 mM). Previous to the analysis, each vial was sonicated (Selecta Ultrasounds bath) for 2 min and the pH was adjusted from values 2–8 by adding either HCl (0.32 and 0.032 M) or NaOH (5 M). The ζ-potential was measured by a Malvern Nano Zetasizer ZS. Data were collected on Zetasizer software. All experiments were run in triplicate.

### Transmission Electron Microscopy

Morphological examinations of the AmyKS–BMNPs and XAn11–MNPs–E/N were carried out using a transmission electron microscope Philips model CM20 equipped with an energy-dispersive X-ray (EDAX) spectrometer. For the visualization, sample drops were placed on copper grids.

### Fourier Transform Infrared Spectroscopy

To further quantify the presence of amylase or xylanase at the surface of the nanoparticles in the performing nanoassemblies, Fourier Transform Infrared (FTIR) absorption spectra of AmyKS–BMNPs and XAn11–MNPs–E/N were determined by FTIR spectroscopy in attenuated total reflection (ATR) mode with a Bruker VERTEX 70/70v model using a KBr disk. Samples were mixed with dry KBr, and then, the mixture was ground to fine powder using an agate mortar before it was compressed into a KBr disk under a hydraulic press at 10,000 psi. Each KBr disk was scanned over a wave number range of 400–4000 cm^–1^.

A direct quantification of the amount of bound AmyKS and XAn11 of the best performing nanoassemblies was done by using FTIR spectroscopy, as stated below. To do that, first, a calibration curve was created by analyzing known amounts of free AmyKS and XAn11 (using the respective buffers as the background), known amounts of BMNPs and MNPs, and mixtures of known amounts of AmyKS + BMNPs and XAn11 + MNPs. The peak height and peak areas were measured by using Spectra Manager Version 2, 2.08.01, JASCO Corporation. Hewlett-Packard (two-points base). The peak intensity at 1634 cm^–1^ for AmyKS and XAn11 and that at 540 cm^–1^ for BMNPs and MNPs from spectra were used for the calibration curves. Peak parameters were compared and plotted against the weight of the relevant sample, yielding the following regression lines
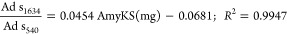
1
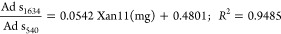
2

By using these equations, the amount of immobilized amylase and xylanase was calculated from the spectra.

### Optimization of the Immobilization. Determination of the Activity

#### Parameters Considered for the Optimization of Enzyme Immobilization

The immobilization process was optimized by choosing, for each enzyme, the substrate and bonding type that yielded the best nanoassembly in terms of immobilized amount and activity and, once that was determined, by varying the initial amount of the free enzyme, the reaction time and, in the case of AmyKS, the concentration of GA used to strengthen the bonding between the enzyme and the substrate.

To optimize the amount of enzyme that could be immobilized on the nanoparticles, different amounts of AmyKS and XAn11 (25, 50, 75, and 100 μM) were used for immobilization onto 5 mg of BMNPs or MNPs. To optimize the minimum concentration of GA needed for the immobilization of AmyKS, different concentrations of GA (0, 0.25, 0.5, and 1%) were tested. The reaction times varied from 6 to 30 h in the case of AmyKS-bearing experiments and from 2 to 10 h in XAn11-bearing ones.

To facilitate the selection process of the best nanoassembly for each enzyme, the amount of immobilized enzyme was indirectly measured by UV–Vis at 280 nm as stated above and the percentage of the immobilized enzyme (immobilization %) was calculated from eq 3:^27^
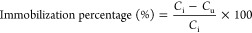
3where *C*_i_ is the initial concentration of the free enzyme used for the immobilization reaction, and *C*_u_ is the concentration of the unbound enzyme after the reaction.

The stability of the nanoassembly was determined by measuring the percentage release (release %) of the immobilized enzyme that is defined as determined in eq 4
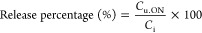
4where *C*_u.ON_ is the concentration of the released enzyme (unbound) and *C*_i_ is the concentration of the enzyme initially used for the reaction.

### Activity Assays of α-Amylase and Xylanase

AmyKS is an α-amylase able to hydrolyze starch on α-d-1,4 glucosidic bonds to produce maltotriose, maltose, and glucose as major final products.^25^ XAn11 is an endoxylanase that catalyzes the hydrolysis of the β-1,4-xylosidic linkages in xylan to produce xylose.^26^ The catalytic activity of nano-biocatalysts was determined by the 3,5-dinitrosalicylic acid (DNS) assay based on the measurement of the reduced sugar released after enzyme hydrolysis.^28^ This method is well established in the area, and many other authors have used the DNS reaction to evaluate the activity of immobilized glycoside hydrolases.^29−31^

The obtained activity of immobilized AmyKS and Xan11 is compatible with an immobilization that does not interfere with the active site of the enzyme(s) and allows the diffusion of the substrate (starch or xylan).^29^

Free AmyKS and AmyKS–BMNPs were suspended in 0.1 M HEPES buffer, pH 7.4. α-Amylase activity was assessed according to the DNS method: 0.5 mL of 1% soluble starch in 0.1 M phosphate buffer (pH 7.0) was added to 0.5 mL of the enzyme suspension and was incubated for 20 min at 70 °C. The reaction was stopped by adding 1 mL of dinitrosalicylic acid reagent and kept in boiling water bath for 10 min.^28^ Absorbance was measured at 540 nm against the enzyme-free blank. One unit of enzyme activity (U) is defined as the amount of enzyme that liberated one mM of the reducing sugar as glucose/min under assay conditions.

The activity of the free XAn11 and XAn11–MNPs–E/N nanoassembly was measured at 50 °C and pH 5. A volume of 0.5 mL of the enzyme solution, diluted in citrate buffer (0.1 M, pH 5), was incubated for 20 min with 0.5 mL of 1% soluble birch wood xylan. The amount of reducing sugars released was determined by the DNS method.^28^ One unit of xylanase activity is defined as the amount of the enzyme that produces 1 μmol of xylose equivalent per minute.

The enzyme activity was calculated according to the following formula
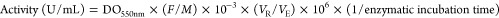
5where *F* is the factor of the DNS reagent, which corresponds to the slope of the standard curve produced with glucose, *V*_R_ is the reaction volume, *V*_E_ is the volume of the test sample, and *M* refers to the glucose molar mass (180 g/mol) for the AmyKS assays and xylose molar mass (150 g/mol) for the XAn11 assays.

#### Activity Percentage and Relative Activity of Immobilized α-Amylase and Xylanase

The activity percentage of immobilized enzyme is defined by the ratio between the activity of the immobilized enzyme (U/mL) and the initial activity of the free enzyme in solution (U/mL). The obtained percentage is referred to the free enzyme present in the solution.

The activity percentage was calculated from eq 6(27)
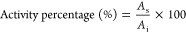
6where *A*_s_ is the activity of the immobilized enzyme (U/mL) and *A*_i_ is the initial activity of the free enzyme in solution before immobilization (U/mL).

The relative activity is calculated to compare the activity of a given sample with the one that yields the maximum activity, to which the value of 100% is assigned for each experiment.

The relative activity of the immobilized enzyme was calculated from eq 7:^29^
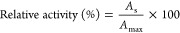
7where *A*_s_ is the activity of the immobilized enzyme of the given sample (U/mL) and (*A*_max_) is the maximum activity of the immobilized enzyme (U/mL) measured in the experiment.

### Storage Stability and Reusability of the Nanoassemblies

The storage stability of immobilized AmyKS was examined by assaying their relative activities after being incubated in phosphate buffer at 4 and 20 °C for a period of 90 and 30 days, respectively. The reusability of the immobilized amylase was determined by testing over 15 cycles. Between each reaction step, the immobilized AmyKS–BMNPs was washed with phosphate buffer (50 mM), pH 7.4. The initial activity was taken as the reference to calculate the percentage activity during each repeated use. All the experiments were performed in triplicate, and the results are expressed as mean values.

In the case of XAn11, the storage stability of the immobilized XAn11 was examined by assaying its relative activity after being incubated in citrate buffer at 4 and 20 °C for a period of 30 and 10 days, respectively. The reusability of the immobilized xylanase was determined by testing over 10 cycles. Between each reaction step, the immobilized XAn11–MNPs–E/N was dissociated from xylose by magnet separation and washed by citrate buffer (50 mM), pH 5. As described earlier, the initial activity was taken as reference to calculate the percentage activity during each repeated use. All the experiments were performed in triplicate, and the results are expressed as mean values.

## Results and Discussion

### α-Amylase and Xylanase Immobilization

The immobilization of AmyKS was tried on the two types of nanoparticles, BMNPs and MNPs, with and without EDC/NHS. Preliminary results show that immobilization of AmyKS on BMNPs (AmyKS–BMNPs) is more effective than that occurring under the other immobilization conditions (Figure S3A). In fact, the immobilization percentage and activity percentage of immobilized enzyme are 71 and 52%, respectively. Indeed, 68% of AmyKS was immobilized on BMNPs–EDC/NHS by retaining only 40% of its initial activity. Results show, also, that the immobilization % and activity % of AmyKS immobilized on MNPs (with and without EDC/NHS) are very low. According to these results, the nanoassembly AmyKS–BMNPs was chosen for further optimization.

Since an enzyme release from BMNPs was detected after overnight of around 31% (Figure S3B), GA was added as a cross-linking reagent to the reaction solution during the immobilization procedure at different concentrations (Table S1). As it is shown in Table S1, the low final concentration of GA (0.25%) allows the highest percentage of immobilized enzyme activity (63%). A slight reduction of activity occurred at GA concentrations above 0.25%, which may be due to the formation of enzyme multimers as a result of the excessive cross-linking.^32,33^ The improvement in the immobilization aimed by GA is consistent with previous studies. In fact, GA is reactive toward lysine residues of proteins and it has been used for protein immobilization through covalent attachment of amino-activated matrixes or by cross-linking of protein–protein aggregates or protein immobilized onto an amino-activated matrix.^34^

**Figure 1:**
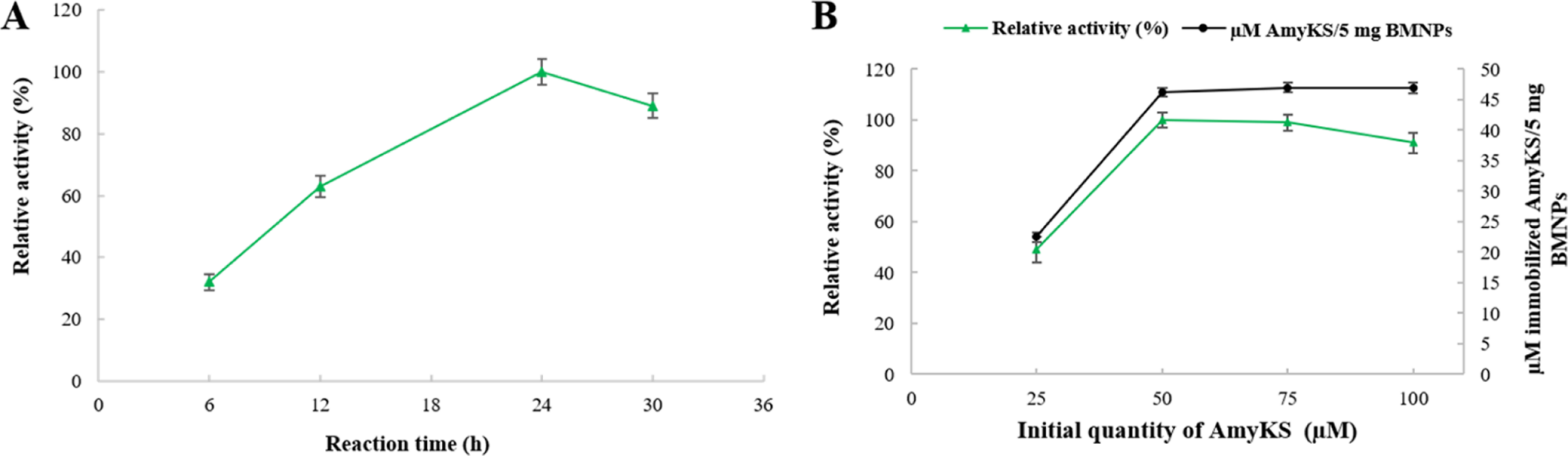
Effect of reaction time and enzyme amount on AmyKS immobilization. (A) Effect of reaction time on the relative activity of immobilized AmyKS and (B) effect of enzyme quantity on the relative activity and on the loading potential of immobilized AmyKS.

Other factors were optimized to maximize the amount of enzyme coupled to BMNPs and the percentage of active AmyKS. For instance, incubation time was found to have an effect on the activity of immobilized AmyKS (Figure 1A). A period of 24 h of incubation resulted in maximum percentage of active immobilized AmyKS (100% of relative activity). To further demonstrate that the measured activity corresponds to that of the immobilized AmyKS–BMNPs and not to that of the free enzyme in solution, a control experiment was performed in which a suspension of AmyKS–BMNPs nanoassemblies was incubated during 20 min at 70 °C without starch. Then, AmyKS–BMNPs were magnetically removed and activity measurements were done on both the immobilized enzyme and the one that could have been released into the supernatant. The result shows that the supernatant does not contain any amylase activity, while all the activity detected corresponds to the nanoassembly AmyKS–BMNPs.

Additionally, compared with different concentrations of AmyKS loaded on BMNPs, results show that 50 μM is the optimal concentration, which promotes the larger activity % of the immobilized AmyKS which is 78% (relative activity 100%; Figure 1B). Based on indirect measurements, the amount of AmyKS immobilized was found to increase by increasing the concentration of enzyme in solution until the BMNPs were saturated. In terms of loading potential, the AmyKS–BMNP nanoassembly was able to carry 46 μM AmyKS per 5 mg of BMNPs (Figure 1B), meaning ∼62.6 wt % AmyKs measured by indirect measurements. This data is comparable to that obtained from direct measurements from the FTIR spectra by applying the corresponding regression equation (71.3 wt % of AmyKs). Enzyme concentrations in solution above 75 μM resulted in loss of activity in the immobilized AmyKS, probably due to steric hindrance,^35^ multilayer formation, or protein aggregation.^36^

After these optimizations, the resulting nanoassembly AmyKS–BMNPs added with 0.25% GA yielded a AmyKS load of 46 μM/5 mg BMNPs (9 μM AmyKS/mg BMNPs) (625 mg/g BMNPs), showing an activity of 78% compared to that of the free enzyme (Table S2).

ζ-Potential values for AmyKS–BMNPs yielded differences with those of BMNPs, showing changes in the surface of the nanoassembly compared to that of unload BMNPs. ζ-Potential values for AmyKS–BMNPs were above zero at pH 2 and below zero from 4 to 8 (Figure S4A). At such, the pI for this nanoassembly is ∼3.0. Therefore, the functionalization switches the isoelectric point from 4.4 for the BMNPs to 3.0 for AmyKS–BMNPs, showing a higher density of negatively charged groups at the nanoassembly surface. This change in the BMNP surface properties indicates the attachment of AmyKS to this surface, and, in fact, this is further confirmed by TEM and FTIR analyses. A layer of less electron-dense material coating the BMNPs can be observed by TEM (Figure S4B,C). FTIR data (Figure S4D) also show absorption bands that are different in BMNPs and AmyKS–BMNPs, and that further confirms the bonding in the nanoassembly. The absorption band at 545 cm^–1^ corresponds to the Fe–O bond of magnetite Fe_3_O_4_ and is evident for both the BMNPs and for the nanoassemblies, although the signal is less intense in the latter, probably due to the shielding because of the protein coating. The peaks at 1656 and 1203 cm^–1^ are correlated with amide I and amide III, respectively, and are consistent with the presence of MamC at the surface of BMNPs.^20^ Interestingly these peaks either do not show (1203 cm^–1^) or are shifted (from 1656 to 1634 cm^–1^) in the nanoassembly, indicating the formation of new bonds (1634 cm^–1^ corresponds to CO–NH^30^) or the shielding of the first ones. The formation of amide bonds is consistent with the cross-linking triggered by GA.^32^

ζ-Potential data and the higher percentage on immobilized AmyKs on BMNPs compared to those on MNPs provide hints regarding the electrostatic binding between BMNPs and AmyKs. Considering the pI of BMNPs (4.4, Figure S4A), MNPs (7.0^20^), and AmyKS (5.61^25^) and that charged domains in AmyKs are exposed to the outer protein surface (thus allowing an electrostatic interaction with the charged nanoparticles surface; Figure 4A,B), different scenarios have to be discussed as a function of the pH of functionalization. At the pH of AmyKS–MNPs formation (pH 5), MNPs were positively charged and so was AmyKS. According to the equilibrium of the Fe-bearing species present on the surface of MNPs in aqueous solutions, at acidic pH, the dominant species at the magnetite surface is Fe(II,III)OH_2_^+^.^37^ Unfortunately, under these conditions, the functionalization was not successful, even in the presence of 1 M Cl^–^ that could mediate the bonding between Fe(II,III)OH_2_^+^ at MNPs and the positively charge residues in AmyKs. Therefore, these positively charged residues do not seem to play an important role in the electrostatic binding.

However, different is the case during the formation of AmyKS–BMNPs. At the functionalization pH (pH 7.4), both BMNPs and AmyKS were negatively charged, but the electrostatic bonding was successful in this case, probably mediated by the presence of 2 M Ca^2+^ that may act as a bridge between the negatively charged domains in BMNPs and AmyKS, hereinafter stabilized by the GA cross-linking. This electrostatic binding is not expected to greatly compromise the activity of the protein if the binding does not occur through the active site, and, as demonstrated by activity measurements, the active site of the protein was not involved in this electrostatic binding.

**Figure 2:**
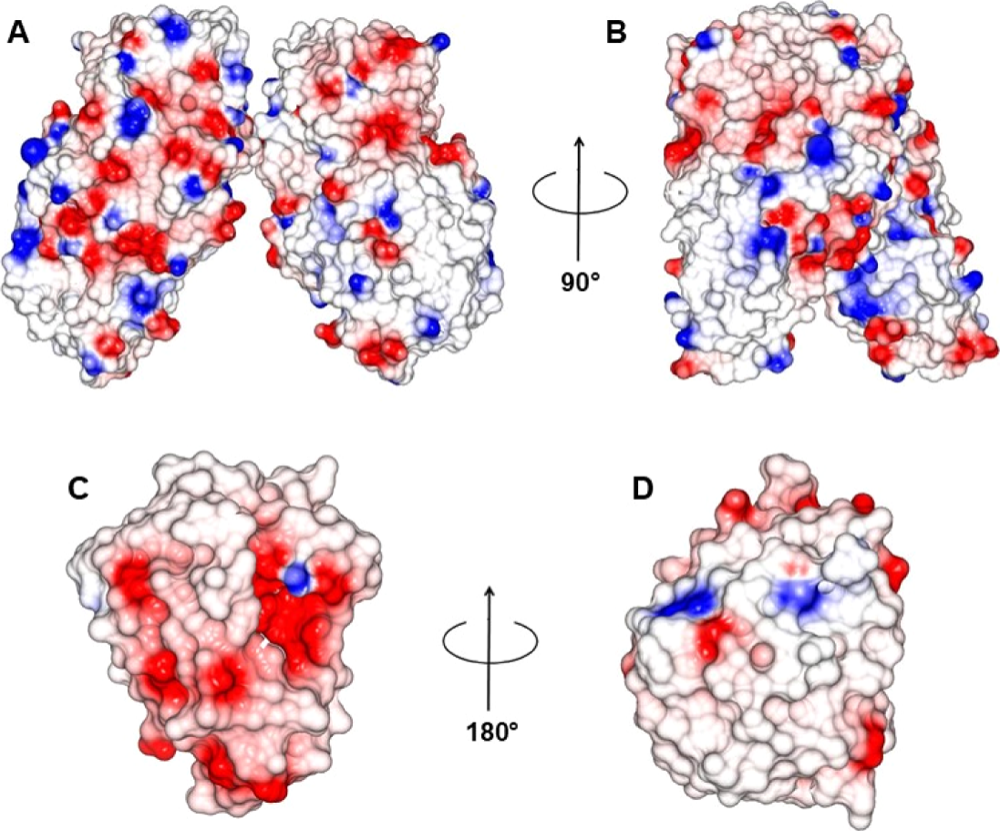
AmyKS and XAn11 surface charge distribution. Molecular surface charge distribution of AmyKS, PDB id: 1BAG, (panel A and B) and XAn11, PDB id: 2QZ2, (panel C and D). The potential distribution was generated and visualized with CCP 4 mg.^38^

On the contrary, different is the scenario in the case of XAn11. As shown in Figure 2C,D, xylanase is not present as there are many charged residues compared to those of AmyKs, and even the conformation of the protein prevents them from being as exposed to the outer as they were in AmyKS. Therefore, according to this model, an electrostatic binding between XAn11 and either BMNPs or even MNPs is not expected. In fact, our results show that the best nanoassembly in terms of immobilization % of XAn11 occurred when this enzyme was bound to MNPs via covalent bonds with EDC/NHS (Figure S5).

**Figure 3:**
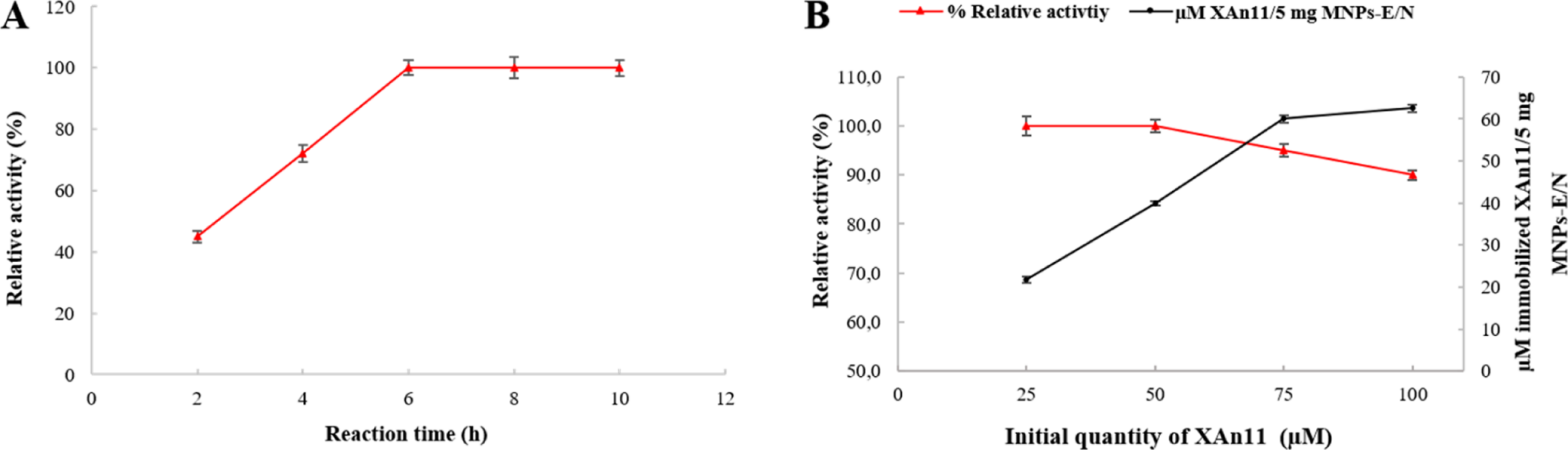
Effect of the reaction time and enzyme amount on XAn11 immobilization. (A) Effect of reaction time on the relative activity of immobilized XAn11 and (B) effect of enzyme quantity on the relative activity and on loading potential of immobilized XAn11.

As with AmyKS, the nanoassembly XAn11–MNPs–E/N was optimized by varying the reaction time (from 2 to 10 h, Figure 3A) and the initial concentration of the protein in solution (Figure 3B). The activity percentage of immobilized XAn11 increased by increasing the reaction time for functionalization from 36.5% (at 2 h) to 81% (at 10 h) compared to the free enzyme (the relative activity: from 45 to 100%). After 6 h of incubation, the relative activity stabilized at 100%, and no significant improvement was observed upon increasing the reaction time, so this 6 h time interval was chosen for further nanoassembly optimization. As before, experiments were performed as indicated above to confirm that the measured activity was that of the immobilized enzyme in the nanoassembly XAn11–MNPs–E/N and not that of the free enzyme in solution that yielded very low xylanase activity (<3%) in the supernatant fraction, while 97% of activity was detected on the fraction containing XAn11–MNPs–E/N.

In terms of the effect of protein concentration in solution on the immobilization % and relative activity % of the immobilized enzyme, our results demonstrate that a maximum relative activity of the nanoassembly (81%) was obtained when the initial concentration of XAn11 in solution was 25 μM (the nanoassemblies carrying 21.8 μM of XAn11 per 5 mg of MNPs), with the relative activity % decreasing at higher concentrations of XAn11 (Figure 3B). As a summary, under the best conditions, the nanoassembly XAn11–MNPs–E/N was able to carry 4 μM XAn11 per mg of MNPs (∼9.08 wt % XAn11), preserving 81% of the activity of the free enzyme (Table S3). This wt % of XAn11 in the nanoassembly, determined from indirect measurements, is comparable to that obtained from direct measurements from the FTIR spectra by applying the relevant regression equation (10.1 wt % XAn11).

As occurring in the case of immobilized AmyKS, the immobilization of XAn11 changed the surface properties of MNPs, according to the ζ-potential data. In fact, the pI for MNPs is pH 7, while the pI for the nanoformulation decreased until pH 4–5 (Figure S6A), a value similar to the pI for free XAn11 (4.31). This result proves the coverage of nanoparticles with this enzyme. These results are further confirmed by TEM images (Figure S6B,C), which show that MNPs are covered by a less electron-dense layer, consistent with the enzyme covering. FTIR data also confirm the presence of XAn11 at the surface of the nanoassembly (Figure S6D). The FTIR spectra of XAn11–MNPs–E/N nanoassemblies show the characteristic absorption peaks of amide groups from xylanase (1520 and 1634 cm^–1^).^39−41^ The band at 2900 cm^–1^ corresponds to the C–H band from xylanase, while the peak at 3300 cm^–1^ is the characteristic of amine (N–H) groups.^41^ In both FTIR spectra (MNP control and XAn11–MNPs–E/N), an absorption band at 548 cm^–1^, which corresponds to the Fe–O bond from magnetite, is observed, confirming the immobilization of the enzyme on MNPs.^41,42^

### Storage Stability of AmyKS–BMNPs and XAn11–MNPs–E/N

**Figure 4:**
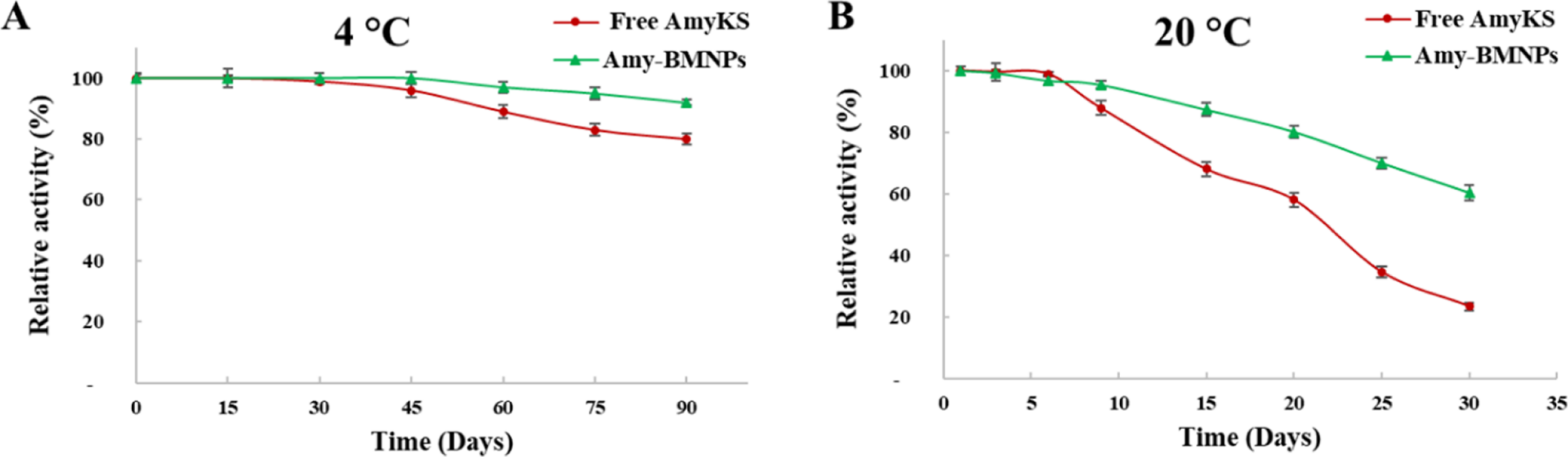
Storage stability comparison between AmyKS–BMNPs and free AmyKS. Storage stability of the AmyKS–BMNPs and free AmyKS, (A) incubation during 3 months at 4 °C and (B) incubation during 30 days at 20 °C.

Free AmyKS and AmyKS–BMNPs maintained more than 80 and 92%, respectively, of their initial activity after 3 months of storage at 4 °C (Figure 4A). After 30 days at 20 °C, the relative activities of free AmyKS and AmyKS–BMNPs were 23.5 and 60%, respectively (Figure 4B). The AmyKS–BMNPs retained significantly more activity compared to that of the free amylase.

These results are in line with those reported by other authors (Table S4), which show an enhancement in the preservation of the enzyme activity upon storage in immobilized versus free enzymes. For instance, Dhavale, et al.^30^ reported that after 20 days at 37 °C, the activities of the free amylase and the amylase immobilized on chitosan-coated MNPs were 18 and 66%, respectively. Sohrabi et al.^43^ have also reported that after 12 days of storage (temperature not indicated), the amylase immobilized on silica-coated Fe_3_O_4_ nanoparticles retained up to 79% activity. The improvement of storage stability can be explained by the rigidity and stability of structure of the immobilized enzyme on the nanoparticle surface.^11^

**Figure 5:**
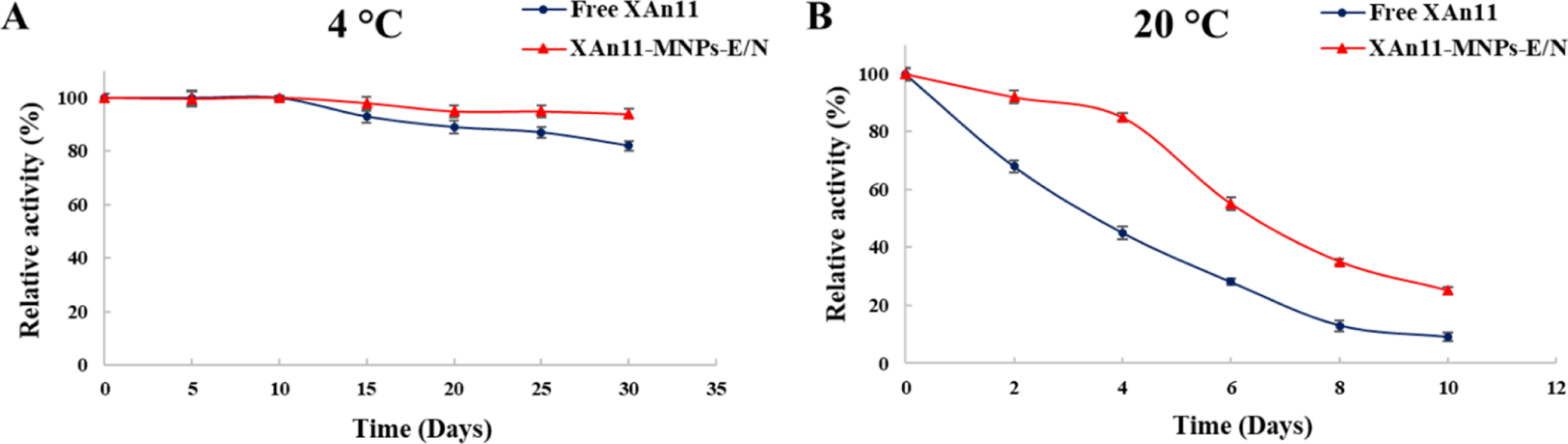
Storage stability comparison between XAn11–MNPs–E/N and free XAn11. Storage stability of the XAn11–MNPs–E/N and free XAn11 (A) incubation during 30 days at 4 °C. (B) Incubation during 10 days at 20 °C.

Identically, XAn11–MNPs–E/N and free XAn11 maintained 85 and 45%, respectively, of their relative activity after 4 days at 20 °C (Figure 5A). Furthermore, XAn11–MNPs–E/N and free XAn11 maintained more than 94 and 72%, respectively, of their relative activity after 30 days of storage at 4 °C (Figure 5B).

These results are in accordance with those observed by Mehnati-Najafabadi et al.^44^ (Table S4), who showed that the storage efficiency after 30 days at 4 °C of free xylanase declined faster than that for xylanase immobilized on MNPs perched on graphene oxide nanosheets. Identically, when xylanase was immobilized on MNP-supported hyperbranched polyglycerol and conjugated with citric acid, the stability of the enzyme increased compared to that of the free enzyme. In fact, the immobilized and free xylanase retained around 95 and 80%, respectively, of their initial activity after 30 days at 4 °C.^27^

### Reusability of the Nanoassemblies AmyKS–BMNPs and XAn11–MNPs–E/N

**Figure 6:**
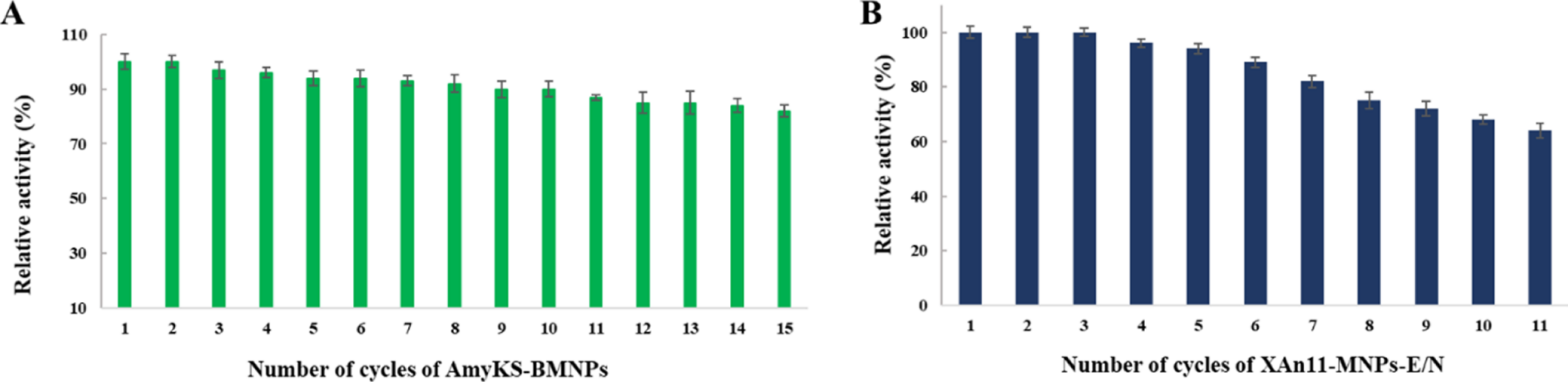
Reusability of the immobilized AmyKS and XAn11. (A) AmyKS–BMNPs were used in 15 subsequent cycles of reaction, and the enzyme still shows up to 82% of its initial activity at the end. (B) XAn11–MNPs–E/N were used in 11 subsequent cycles of reaction, and the enzyme still shows up to 64% of its initial activity at the end.

The increase in the activity of the enzyme over time is definitely one of the advantages of immobilization, but, being the magnetic carrier, a crucial advantage of these particular nanoassemblies is the potential for their magnetic recovery upon enzyme recycling for further use. The reusability of AmyKS–BMNPs is shown in Figure 6A, which demonstrates that the activity of the AmyKS in the nanoassembly only slightly decreased after each run. The AmyKS–BMNPs were reused for 15 cycles, and the enzymes retained up to 82% of their initial activity. This performance is higher than that shown in other studies. For instance, according to Baskar et al.,^45^ only 65% of the initial activity of the enzyme can be detected after six successive hydrolyses run in a nanoassembly formed by an α-amylase immobilized on MNPs via covalent bonds. Defaei et al.^46^ reported also that α-amylase immobilized onto naringin- functionalized MNPs could still retain around 50% of its initial activity after 10 reaction cycles. These results show that BMNPs are potential biotechnological candidates for immobilizing amylases that could be of biotechnological interest, improving enzyme stability both in short and long term and allowing the recycling of the enzyme, showing that these nanoassemblies perform better than the others produced earlier.

In the context of XAn11 immobilization, our results show that the nanoassembly XAn11–MNPs–E/N can also be easily separated from the reaction mixture by means of a magnet and reused afterward for the hydrolysis of xylan in several runs. The reusability of XAn11–MNPs–E/N was assayed over 11 reaction cycles under the optimal assay conditions at pH 5 and 50 °C. A gradual loss in activity was observed after 5 cycles (94% at cycle 5, Figure 6B). The relative activity after 11 cycles was ∼64% of the original. Our results are in agreement with previous studies of xylanases immobilized on other nanoparticles. According to Pal and Khanum,^47^ xylanase immobilized on alginate beads could be reused 5 times while retaining more than 85% of its original activity. Also Soozanipour et al.^39^ reported that the xylanase immobilized on MNPs could retain 65% of its initial activity after 8 cycles. Still, it is noticeable that the nanoassembly produced in the present study yields better performance than those proposed in previous studies.

## Conclusions

The results from the present study demonstrate that MNPs can be used as an attractive and innovative matrix for immobilizing α-amylase and xylanase enzymes to improve their storage stability and enhance their stability over several reaction runs. Furthermore, thanks to the magnetic properties of the nanoassemblies, the immobilized α-amylase and xylanase can be easily recovered from the reaction mixture, avoiding an expensive purification process or, worse, having to inactivate the enzyme before product commercialization, and conveniently reuse them for a new enzymatic reaction. BMNPs have shown to be promising substrates for enzyme immobilization for the first time. The results in the present study show that our nanoassemblies are potential candidates to improve the stability and the recycling of amylases and xylanases of biotechnological interest, showing better performance that other nanoassemblies produced earlier.

## Supporting Information Available

Effect of GA concentration on AmyKS immobilization and activity, AmyKS immobilization efficiency, XAn11 immobilization efficiency, comparison of the present results with literature data functionalization reaction for AmyKS, functionalization reaction for XAn11, AmyKS immobilization and activity, physicochemical characterization of AmyKS–BMNPs, XAn11 immobilization and activity, and physicochemical characterization of XAn11–MNPs–E/N (PDF)

## Abbreviations

MNPs: magnetite nanoparticles
BMNPs: biomimetic magnetic nanoparticles
AmyKS: α-amylase
XAn11: xylanase
MamC: magnetosome-associated protein
EDC: 1-ethyl-3-(3-dimethylaminopropyl) carbodiimide
NHS: *N*-hydroxysuccinimide
GA: glutaraldehyde
AmyKS–BMNPs: AmyKS immobilized on BMNPs
XAn11–MNPs–E/N: Xan11 immobilized on MNPs by EDC/NHS
Fe_3_O_4_: magnetite
c-Fe_2_O_3_: magnetite
pI: isoelectric point
XRD: powder X-ray diffraction
TEM: transmission electron microscopy
EDAX: energy-dispersive X-ray spectrometer
FTIR: Fourier transform infrared
ATR: attenuated total reflection
DNS: dinitrosalicylic acid
U: enzyme activity
