# Supplementary material for: Enzyme Storage and Recycling: Nanoassemblies of α-Amylase and Xylanase Immobilized on Biomimetic Magnetic Nanoparticles
Source: ACS Sustain Chem Eng. 2021 Mar 9;9(11):4054–63. doi: 10.1021/acssuschemeng.0c08300 (PMC8765010; doi:10.1021/acssuschemeng.0c08300)
Supplement: Supplementary file 1 — sc0c08300_si_001.pdf [file sc0c08300_si_001.pdf]

Supplementary Information for

Enzyme storage and recycling: nanoassemblies of α-amylase and xylanase immobilized on biomimetic magnetic nanoparticles

Karima Salem[1], Ylenia Jabalera[2], Jose David Puentes-Pardo[2], Jesus Vilchez-Garcia[2], Adel Sayari[3], Aïda Hmida-Sayari[1], Concepcion Jimenez-Lopez[2,], Massimiliano Perduca[4,*]*

[1]Centre de Biotechnologie de Sfax (CBS), Université de Sfax, Route de Sidi Mansour Km 6, BP "1177" 3018 Sfax, Tunisie

[2]Departamento de Microbiologia, Universidad de Granada, Campus de Fuentenueva s/n, 18071, Granada, Spain.

[3]ENIS, Université de Sfax BP "1173",3038 Sfax, Tunisie

[4]Department of Biotechnology, University of Verona, Strada Le Grazie 15, 37134 Verona, Italy

*Corresponding authors: Concepcion Jimenez-Lopez, Massimiliano Perduca
Email: cjl@ugr.es; massimiliano.perduca@univr.it

Number of pages: S1-S13
Number of Tables: Table S1-S4
Number of Figures: Figure S1-S6

Table S1. Effect of glutaraldehyde concentration on AmyKS immobilization and activity. The effect of GA concentration on the percentage of AmyKS immobilized referred to that initially present in solution, and percentage of activity of AmyKS-BMNPs referred to that of the free enzyme measured under identical concentration as that used for the immobilized one.

**GA concentration (%)**	**0**	**0,25**	**0,5**	**1**
**Immobilization (%)**	49.0 ±0.9	83.0 ±0.5	83.0 ±0.3	82.0 ±0.2
**Activity (%)**	31.0 ±1.5	68.0 ±1.3	66.0 ±1.5	64.5 ±2.3

Table S2. AmyKS immobilization efficiency. Percentage of AmyKS immobilized [(1)] referred to that initially present in solution, percentage of activity of AmyKS-BMNPs [(2)] referred to that of the free enzyme measured under identical concentration as that immobilized, relative activity [(3)] and loading potential.

Immobilization efficiency	Values
**Activity % of AmyKS-BMNPs (%)**	78.00 ±0.16
**Relative activity of AmyKS-BMNPs (%)**	100.0 ±0.2
**Immobilization % (%)**	92.0 ±0.7
**μM AmyKS/5 mg BMNPs**	46.00 ±0.35
**mg AmyKS/ g BMNPs**	625.6 ±4.7

(1) The percentage of AmyKS-BMNPs is the ratio between the concentration of AmyKS-BMNPs and the concentration of AmyKS initially present in solution.

(2) The percentage of activity of AmyKS-BMNPs is the ratio between the activity of AmyKS-BMNPs and the activity of AmyKS initially present in solution.

(3) The relative activity is the ratio between the activity of AmyKS-BMNPs measured under such condition in an experiment and the maximal activity of AmyKS-BMNPs measured in the experiment.

Table S3. XAn11 immobilization efficiency. Percentage of XAn11 immobilized (1) referred to that initially present in solution, percentage of activity of XAn11-MNPs-E/N (2) referred to that of the free enzyme measured under identical concentration as that immobilized, relative activity (3) and loading potential.

Immobilization efficiency	Values
**Activity % of XAn11-MNPs-E/N (%)**	81.0 ±1.9
**Relative activity of XAn11-MNPs-E/N (%)**	100.0 ±2.4
**Immobilization % of XAn11-MNPs-E/N (%)**	87 ±2
**µMol XAn11/5 mg MNPs-E/N**	21.8±0.5
**mg XAn11/ g MNPs-E/N**	90.8 ±2.4

(1) The percentage of XAn11-MNPs-E/N is the ratio between the concentration of XAn11-MNPs-E/N and the concentration of XAn11 initially present in solution.

(2) The percentage of activity of XAn11-MNPs-E/N is the ratio between the activity of XAn11-MNPs-E/N and the activity of XAn11 initially present in solution.

(3) The relative activity is the ratio between the activity of XAn11-MNPs-E/N measured under such condition in an experiment and the maximal activity of XAn11-MNPs-E/N measured in the experiment.

Table S4. Comparison of the present results with literature data.

	Immobilization method and support	% of immobilized enzyme	Loading capacity	Storage efficiency of immobilized enzyme	Storage efficiency of free enzyme	Reusability of immobilized enzyme	References
**Xylanase**	Styrene-divynilbenzene adsorbent resin, with glutaraldehyde	42%	-	-	-	~**70%** after **11** cycles	*Kapoor, et al., 2007*
	Oxide nanosheets polyethylene glycol bis amine (PEGA)	58%	445µg/mg nanocomposite	**35%** after 90 days at 4 ºC	**20%** after 90 days at 4°C	**40%** after **8** cycles at 60°C	*Mehnati-Najafabadi et al., 2017*
	Superparamagnetic graphene oxide nanocomposite		290µg/mg nanocarrier	**60%** after 90 days at 4°C	**35%** after 90 days at 4°C	**50%** after **4** cycles	*Mehnati-Najafabadi et al., 2019*
	Magnetic Nanoparticle/ EDC-NHS	81%	90 mg/g MNPs	**94%** after 30 days at 4°C	**72%** afetr 30 days at 4°C	**64 %** after **11** cycles 50°C	*Present Work*
**Amylase**	Gum acacia - magnetite nanoparticles using glutaraldehyde	60%	0.6 mg/g nanocarrier	-		**70%** after 6 cycles	*Swarnalatha et al., 2013*
	Nano pore zeolite for covalent attachment	58.44%	60µg/100mg nanocarrier	**80%** after 15 days at 4°C	A **total loss** of activity after 15 days at 4°C	-	*Talebi al., 2016*
	Polycaprolactone-grafted magnetic nanoparticles	61%	158µg/mg nanocarrier	~**60%** after 30 days at 4°C	**~40 %** after 90 days at 4°C	>**50%** after **5** cycles	*Defaei et al., 2020*
	Chitosan coated Fe3O4 MNPs (AMNPs)	78%	-	**66 %** after 20 days at 37°C	**18%** after 20 days at 37°C	**79%** after 20 cycles	*Dhavale et al., 2018*
	Silica-coated modified magnetite nanoparticles	45%	-	**79%** after 12 days at 4°C	**40.61%** after 12 days at 4°C	**85.22%** of after 6 cycles	*Sohrabi et al., 2014*
	Biomimetic magnetic nanoparticles/ glutaraldehyde	92%	625 mg/g BMNPs	**92%** after 90 days at 4°C	**80%** after 90 days at 4°C	**82%** after 15 cycles	*Present work*

Figure S1. Functionalization reaction for AmyKS. Representative scheme of the functionalization reaction of AmyKS with BMNPs by electrostatic interaction using glutaraldehyde as crosslinking agent.

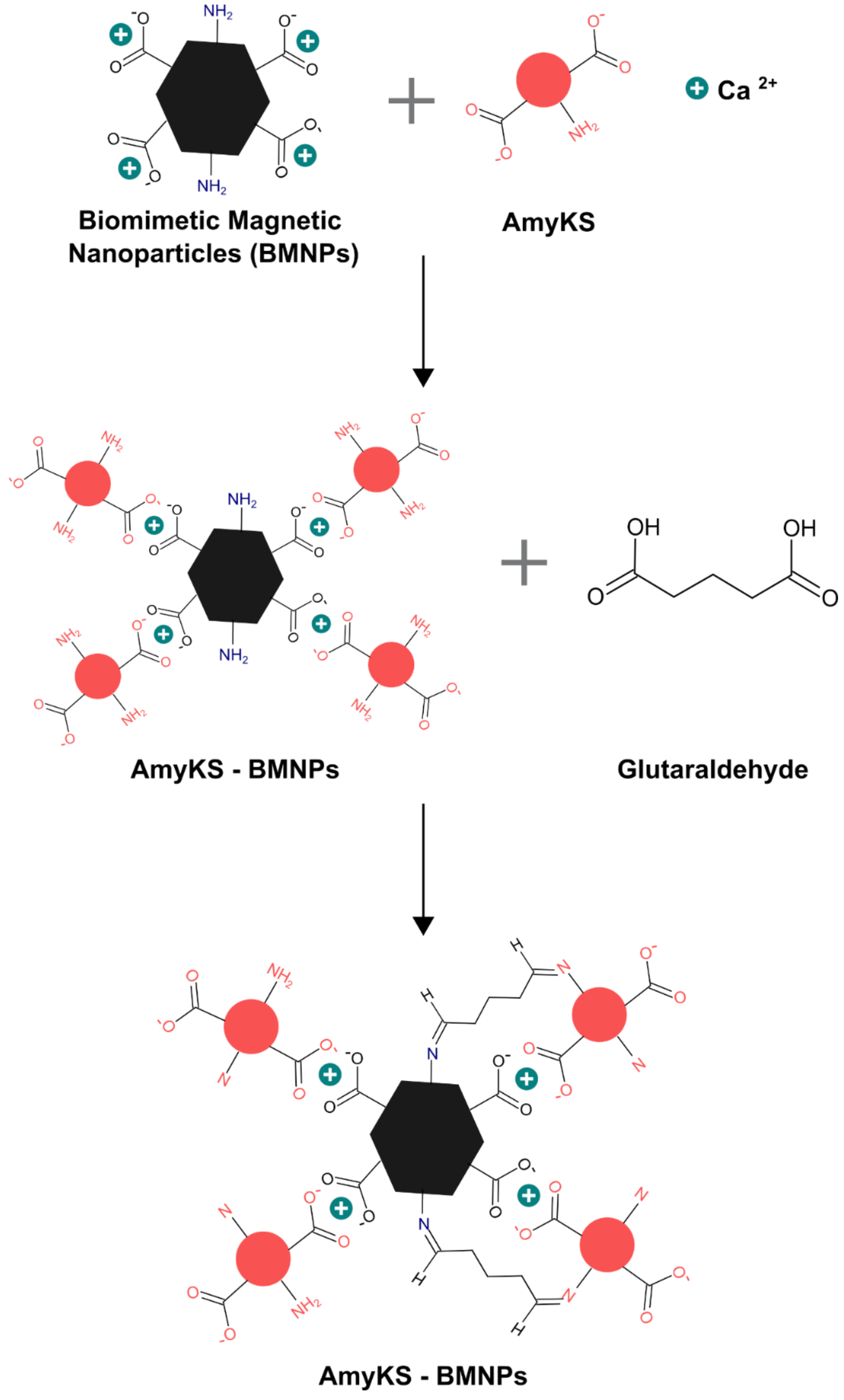

Figure S2. Functionalization reaction of XAn11. Representative scheme of the functionalization reaction of XAn11 with MNPs by covalent bond using EDC/NHS as coupling agents.

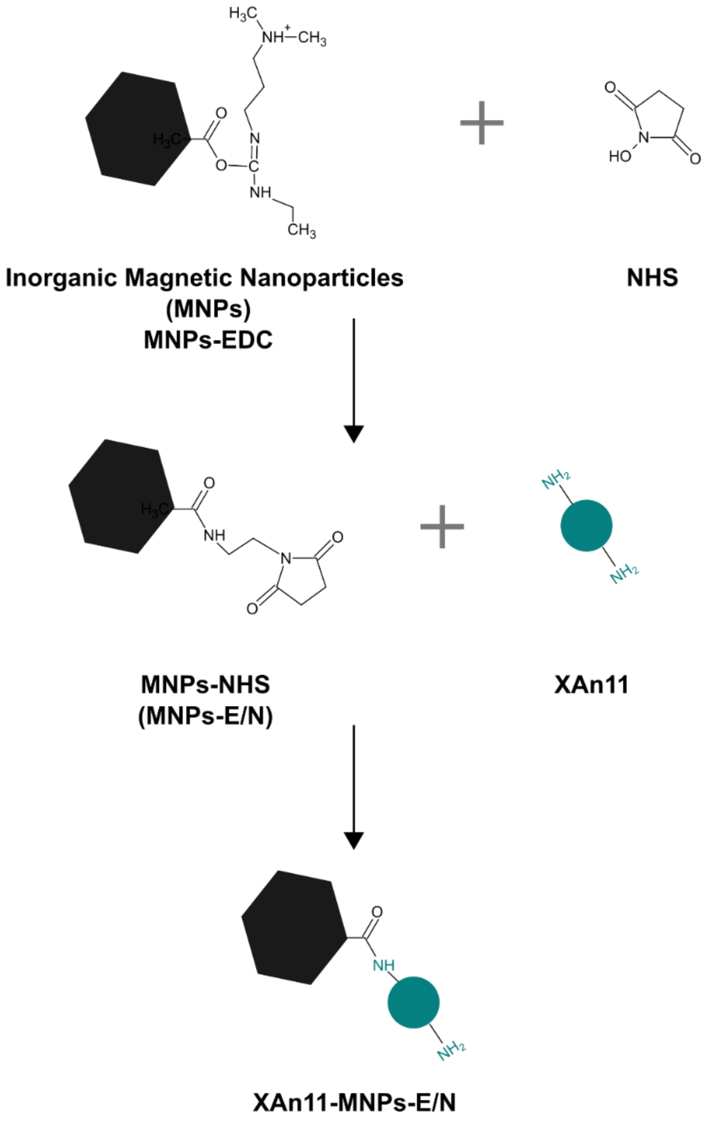

Figure S3. AmyKS immobilization and activity. (A) Results of AmyKS immobilization % and activity % for the four different strategies used to produce the nanoassemblies, (B) AmyKS residual immoblization % and activity % for the optimal nanoassembly right after immobilization (T0) and after overnight incubation.

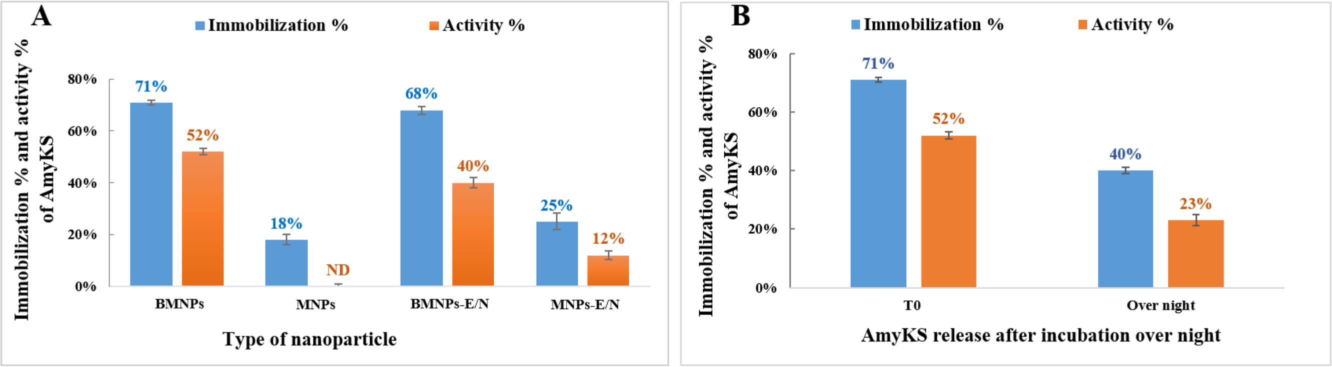

Figure S4. Physico-chemical characterization of AmyKS-BMNPs. (A) ζ-potential of immobilized AmyKS on Biomimetic Magnetic Nanoparticles (AmyKS-BMNPs), (B) TEM image of AmyKS-BMNPs, (C) TEM image of BMNPs and (D) FT-IR spectra of BMNPs and AmyKS-BMNPs. Scale bar corresponds to 100 nm. Black arrows indicate a less electron dense layer covering the magnetic nanoparticles.

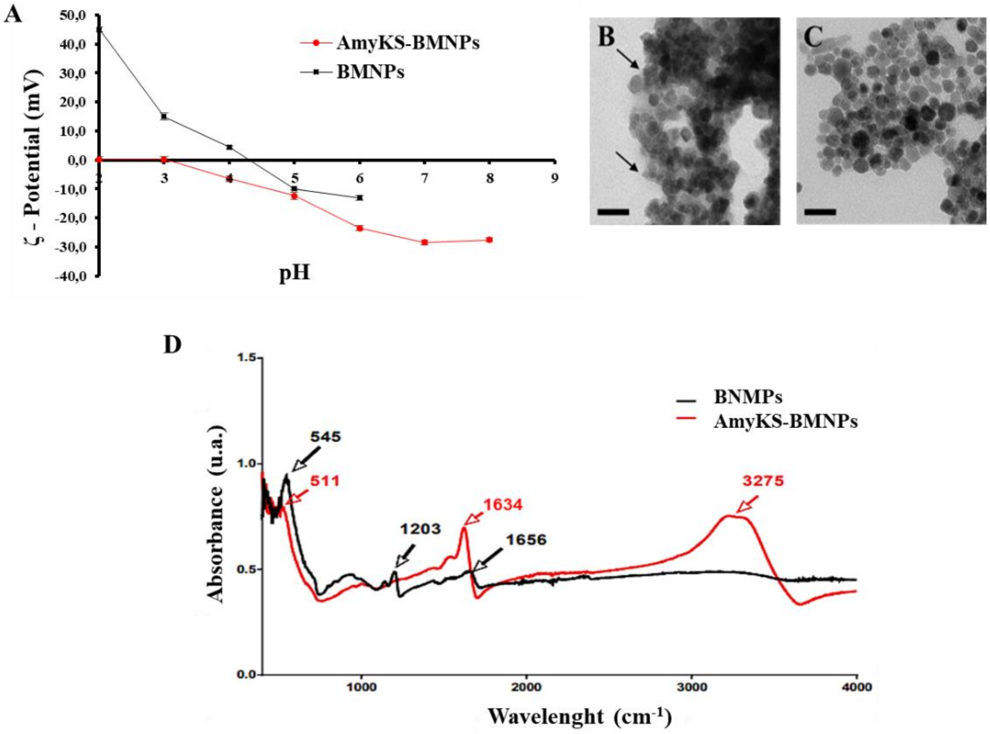

Figure S5. XAn11 immobilization and activity. Results of XAn11 immobilization % and activity % for the four different strategies used to produce the nanoassemblies.

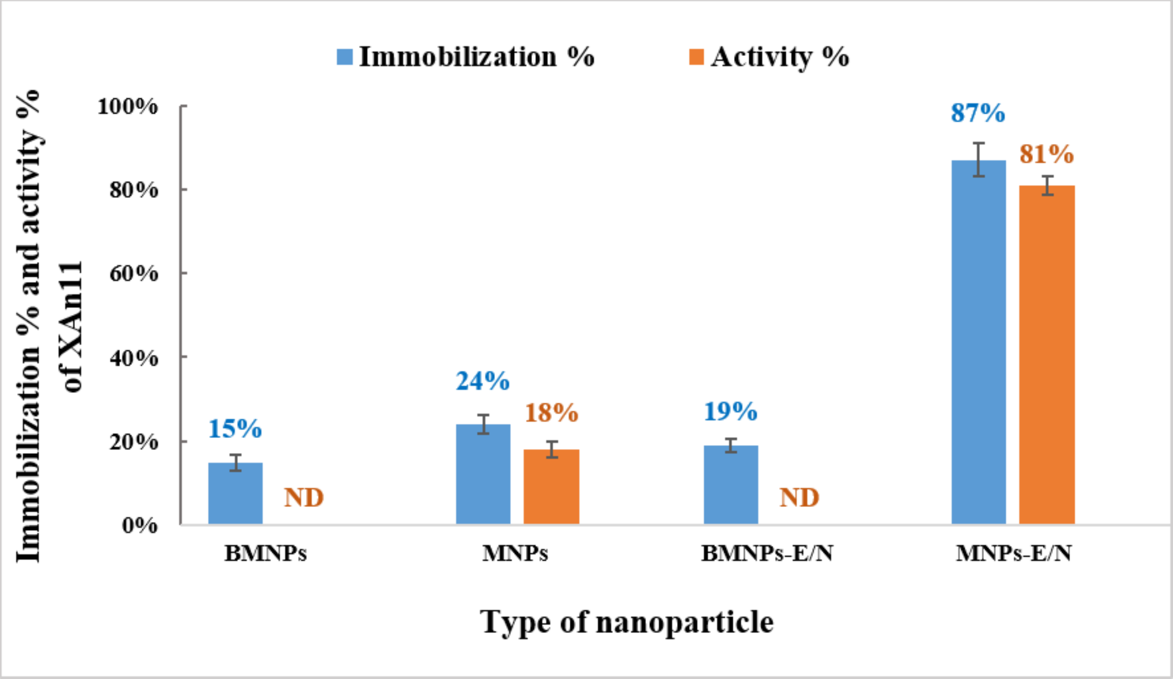

Figure S6. Physico-chemical characterization of XAn11-MNPs-E/N. (A) ζ-potential of immobilized XAn11 on MNPs, (B) TEM image of XAn11-MNPs-E/N, (C) TEM image of MNPs and (D) FT-IR spectra of MNPs and XAn11-MNPs-E/N**.** Scale bar corresponds to 100 nm. Black arrows indicate a less electron dense layer covering the magnetic nanoparticles.

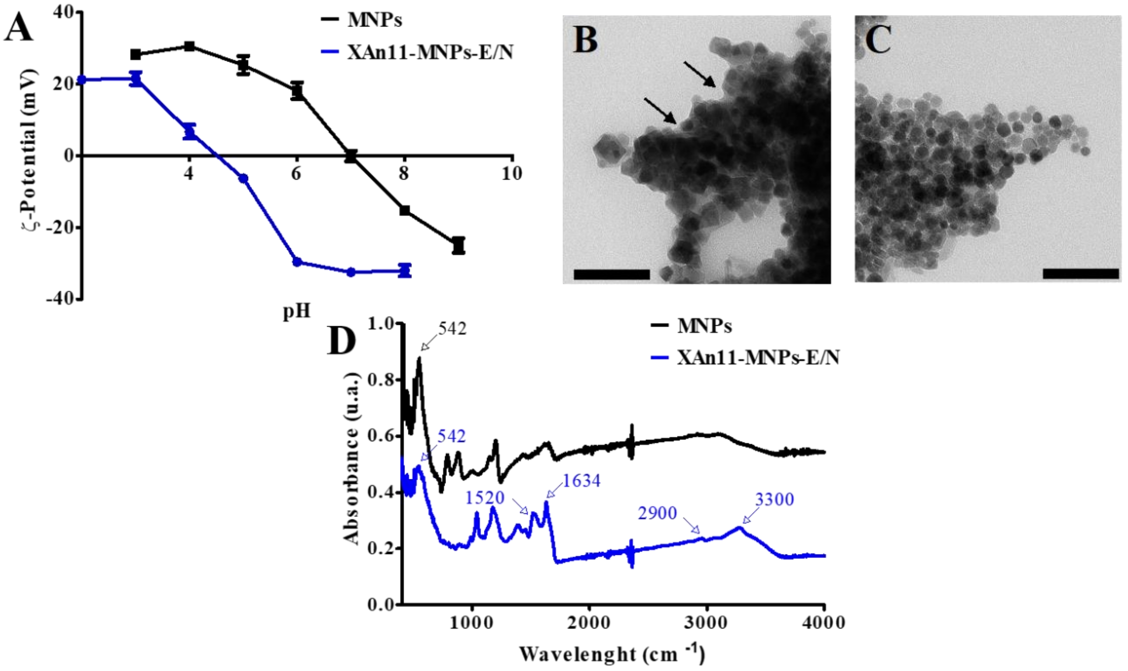

References:

Kapoor, M.; Kuhad, R. C. Immobilization of Xylanase from Bacillus Pumilus Strain MK001 and Its Application in Production of Xylo-Oligosaccharides. *Appl Biochem Biotechnol* **2007**, *142* (2), 125–138. https://doi.org/10.1007/s12010-007-0013-8.

Mehnati-Najafabadi, V.; Taheri-Kafrani, A.; Bordbar, A.-K. Xylanase Immobilization on Modified Superparamagnetic Graphene Oxide Nanocomposite: Effect of PEGylation on Activity and Stability. *International Journal of Biological Macromolecules* **2018**, *107*, 418–425. https://doi.org/10.1016/j.ijbiomac.2017.09.013.

Mehnati-Najafabadi, V.; Taheri-Kafrani, A.; Bordbar, A.-K.; Eidi, A. Covalent Immobilization of Xylanase from Thermomyces Lanuginosus on Aminated Superparamagnetic Graphene Oxide Nanocomposite. *J IRAN CHEM SOC* **2019**, *16* (1), 21–31. https://doi.org/10.1007/s13738-018-1477-x.

Swarnalatha, V.; Aluri Esther, R.; Dhamodharan, R. Immobilization of α-Amylase on Gum Acacia Stabilized Magnetite Nanoparticles, an Easily Recoverable and Reusable Support. *Journal of Molecular Catalysis B: Enzymatic* **2013**, *96*, 6–13. https://doi.org/10.1016/j.molcatb.2013.05.022.

Talebi, M.; Vaezifar, S.; Jafary, F.; Fazilati, M.; Motamedi, S. Stability Improvement of Immobilized A-Amylase Using Nano Pore Zeolite. *Iran J Biotechnol* **2016**, *14* (1), 33–38. https://doi.org/10.15171/ijb.1261.

Defaei, M.; Taheri-Kafrani, A.; Miroliaei, M.; Yaghmaei, P. Alpha-amylase Immobilized on Polycaprolactone-grafted Magnetic Nanoparticles: Improving Stability and Reusability. *J Chem Technol Biotechnol* **2020**, *95* (8), 2243–2250. https://doi.org/10.1002/jctb.6412.

Dhavale, R. P.; Parit, S. B.; Sahoo, S. C.; Kollu, P.; Patil, P. S.; Patil, P. B.; Chougale, A. D. α -Amylase Immobilized on Magnetic Nanoparticles: Reusable Robust Nano-Biocatalyst for Starch Hydrolysis. *Mater. Res. Express* **2018**, *5* (7), 075403. https://doi.org/10.1088/2053-1591/aacef1.

Sohrabi, N.; Rasouli, N.; Torkzadeh, M. Enhanced Stability and Catalytic Activity of Immobilized α-Amylase on Modified Fe_3O_4 Nanoparticles. *Chemical Engineering Journal* **2014**, *240*, 426–433. https://doi.org/10.1016/j.cej.2013.11.059.
